# Short-term bridging and bird-dog exercise programs did not enhance trunk performance and whole-body dynamic balance in young physically active males: A double-blind randomized trial

**DOI:** 10.1371/journal.pone.0325040

**Published:** 2025-06-05

**Authors:** Amaya Prat-Luri, Francisco J. Vera-Garcia, Pedro Moreno-Navarro, Casto Juan-Recio, Javier de los Ríos-Calonge, Juan R. Heredia-Elvar, Jose L. L. Elvira, David Barbado

**Affiliations:** 1 Sports Research Centre, Department of Sport Sciences, Miguel Hernández University, Elche, Spain; 2 Institute for Health and Biomedical Research (ISABIAL Foundation), Miguel Hernández University of Elche, Elche, Spain; 3 Department of Physical Activity and Sports Science, Alfonso X El Sabio University, Madrid, Spain; Universidade de Aveiro Escola Superior de Saude de Aveiro, PORTUGAL

## Abstract

Bridges and bird-dog exercises are commonly used in general training, as well as in warm-up and cool-down routines for young athletes to boost performance and prevent injuries. They are frequently paired with limb and other trunk exercises, and performed without precise control over intensity, which hinders the understanding of their actual impact. This double-blinded randomized controlled trial aimed to evaluate the effects of two bridging and bird-dog exercise programs (one emphasizing intensity, the other volume) on trunk performance and whole-body balance. Sixty participants were randomly assigned to a control group and two experimental groups, both of which performed bridging and bird-dog exercises at a specific intensity controlled by a smartphone-accelerometer. The exercises were conducted twice a week for six weeks at the university sports complex. The effects were assessed on: (i) trunk stability, through the bridging and the bird-dog lumbopelvic postural control, the unstable sitting and the sudden loading sitting tests, (ii) trunk endurance, through the front and the dominant side bridge endurance, and the Biering-Sorensen tests, and (iii) whole-body dynamic balance, through the Y-Balance, the tandem and single-leg stance, and the single-leg triple hop tests. Pre-post changes were reported in both absolute (Δ) and relative (Δ%) values. A two-way mixed ANOVA assessed differences between experimental and control groups, while paired t-tests analyzed within-group pre-post changes with a significance level set at *p* < 0.05. Neither of the experimental groups showed improvements in trunk performance and balance compared to the control group or among themselves. Nonetheless, the higher intensity group elicited greater pre-post changes in the bridging and the bird-dog lumbopelvic postural control tests (−10.4 ≤Δ% ≤−16.9 vs −4.8 ≤Δ% ≤−13.6), whilst the higher volume group did in the trunk endurance tests (10.9 ≤Δ% ≤19.5 vs 7.1 ≤Δ% ≤15.5). The lack of significant between-group differences may be due to the low exercise doses, typical for these exercises in fitness and rehabilitation routines, and the participants being active young males with no apparent postural control deficits. Additionally, the pre-post changes in the experimental groups highlight the specificity of exercise adaptations. This study questions the effectiveness of bridging and bird-dog exercises for improving trunk performance and whole-body balance in this population, beyond the tasks used in training.

## Introduction

Bridges and bird-dogs are some of the most used exercises to train the trunk and core muscles. They have shown to effectively activate trunk and core muscles while challenging the individuals’ ability to maintain the spine in a neutral position while imposing minimal back compressive loads. Additionally, as floor-based movements, they offer easy progressions while keeping the emphasis on trunk and core engagement. Their minimal equipment requirements make them highly accessible and independent of specialized gear [1]. These exercises are considered helpful not only to improve trunk performance-related outcomes (mainly trunk stability and endurance) but also for the improvement of motor performance during athletic or sports activities with high balance demands (e.g., jumping, landing, cutting manoeuvres…) [2,3]. Due to this, bridging and bird-dog exercises are frequently included into young athletes’ workout routines to boost their athletic performance and prevent injuries in the lower back and lower limbs [2].

Despite their perks and popularity, there are some drawbacks that make the positive impact that bridging and bird-dog exercise routines might have on trunk performance and balance in young male athletes difficult to confirm. The main limitation stems from the fact that these exercises are not the only exercises carried out as they are usually combined with other trunk-focused exercises (e.g., crunches, sit-ups, back extensions…) and/or general exercises in which the lower limbs are highly involved (e.g., lunges, squats, balance from standing position…) [4–6]. Another significant limitation of the programs that include bridging and bird-dog exercises lies in the scant analysis of their impact on trunk performance, even though these exercises focus on the trunk. Furthermore, most of the specific literature that does evaluate the effect on trunk performance examines the impact of these programs on trunk endurance [2], although there is limited evidence on how they affect other capabilities such as trunk stability, which has been linked with athletic performance improvement and back and lower limb injury risk reduction [7–10]. Moreover, floor-based exercise programs generally lack a precise monitoring and control of the training load, which makes it difficult to know what doses of these exercises could be most effective. In this sense, conversely to volume parameters [3], no experimental study has objectively quantified to date the bridging and bird-dog exercise intensity. Based on all these limitations, and despite the widespread use of these exercises in fitness, sports and rehabilitation, there is no adequate knowledge of their real impact in young individuals.

In light of the aforementioned, this study aimed to explore the effects of two 6-week bridging and bird-dog exercise programs on trunk performance (i.e., stability and endurance) and whole-body dynamic balance in physically active young males. Both programs consisted of performing exclusively bridging and bird-dog exercise variations: one program using higher intensity exercise variations and another one using longer duration exercise variations. The trunk stability and endurance were assessed during the exercise itself which was carried out, or during similar bridging and bird-dog training exercises [11]. Trunk stability and whole-body dynamic balance were evaluated through well-known biomechanical and field tests [11] that have been related with back and lower limb injury risk [7–10,12–14]. Considering the results of a recent correlational study in young physically active males showing no association of the lumbopelvic postural control tests with the rest of the aforementioned trunk stability and whole-body dynamic balance tests [11], it was hypothesized that both bridging and bird-dog exercise programs would not have a significant impact on these tests. On the other hand, based on the trunk training and testing specificity [15], these exercise programs would have a significant impact on the lumbopelvic postural control and the trunk muscle endurance tests. In this sense, it was also hypothesized that the higher intensity exercise program would evoke greater effects on the lumbopelvic postural control tests, and the higher volume exercise program would induce a greater impact on the trunk muscle endurance tests.

## Methods

### Participants

Sixty-three healthy males were recruited between the dates of 01/09/2017 and 31/09/2018. They were physically active, practicing 2–5 sessions per week of 30–120 min of light to vigorous physical exertion. Participants were excluded if they: i) were high-performance athletes whose sport modality required high demands of trunk performance (e.g., judokas or gymnasts) and therefore they might exhibit higher levels of trunk performance compared to the general population of physically active males that could potentially bias the results; ii) followed a structured training program targeting the trunk structures; or iii) presented a disease that contraindicated performing physical exercise (e.g., musculoskeletal injuries, coronary diseases, visual or vestibular problems, etc.). Prior to the beginning of the study, all the participants filled in an informed consent. The study protocol was approved by the University Office for Research Ethics (DPS.FVG.02.14). Likewise, the individuals featured in this manuscript have provided written informed consent, as per the PLOS consent form, granting permission to publish the images in which they appear.

### Experimental design and randomisation

This study was a double-blinded randomized controlled trial (ClinicalTrials.gov: registration reference: NCT03459430) paired according to the participants’ initial trunk stability level registered through the different lumbopelvic postural control tests. Participants were randomly assigned to an experimental group which performed the higher intensity exercise program (EG_HI_), an experimental group which performed the higher volume exercise program (EG_HV_) or a control group (CG), in which participants did not train and continued with their regular activity. Participants were randomized by an independent researcher through opaque envelopes and informed not to start any new exercise program during the study period. There were no deviations from the registered protocol.

### Testing protocols

All the participants followed two weeks of physical assessments before the training period (pre-test) and one week of physical assessments after the training period (post-test). Due to the high number of trials performed, each assessment week comprised two testing sessions spaced by a one-day rest. The lumbopelvic postural control tests were recorded in the first testing session, whilst the rest of the testing protocols were performed in the second testing session. These assessments were performed by the same blinded researchers for all the groups, who were not involved in the intervention. Although all the tests used in this study have been comprehensively reported by de los Ríos-Calonge et al. [11] they are briefly described below:

#### Trunk stability tests.

**Fig 1:**
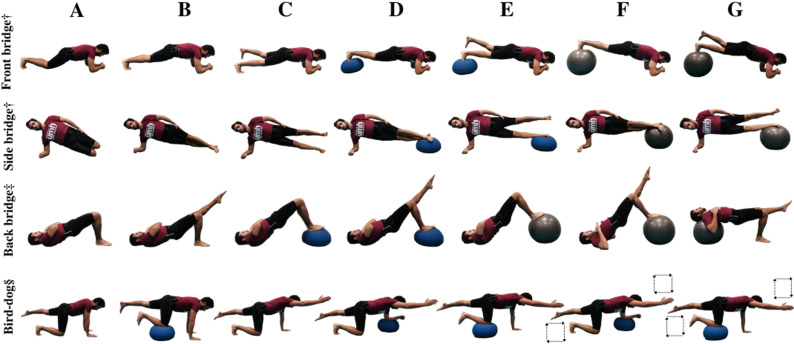
Bridging and bird-dog exercises. †Front and side bridge variations: A) short bridge; B) long bridge; C) bridging with single leg support; D) bridging with double leg support on a hemisphere ball; E) bridging with single leg support on a hemisphere ball; F) bridging with double leg support on a fitball; G) bridging with single leg support on a fitball. ‡Back bridge variations: A) short bridge; B) bridging with single leg support; C) bridging with double leg support on a hemisphere ball; D) bridging with single leg support on a hemisphere ball; E) bridging with double leg support on a fitball; F) bridging with single leg support on a fitball; G) bridging with single leg support and with the upper back on a fitball. §Bird-dog variations: A) three-point position with an elevated leg; B) three-point position with the knee on a hemisphere ball; C) classic two-point bird-dog position with elevated contralateral leg and arm; D) two-point bird-dog position with the forearm on a hemisphere ball; E) two-point bird-dog position with the knee on a hemisphere ball; F) two-point bird-dog position with the forearm on a hemisphere ball while drawing squares in the air with the elevated limbs; G) two-point bird-dog position with the knee on a hemisphere ball while drawing squares in the air with the elevated limbs.

(i) the lumbopelvic postural control tests were performed to assess the lumbopelvic postural control during seven 15-s variations of front bridge, back bridge, dominant side bridge and bird-dog exercises (Fig 1), based on the lumbopelvic accelerations (m/s^2^) measured using a free mobile application (Accelerometer Analyzer, Mobile Tools, Sopot, Poland) with a tri-axial smartphone-accelerometer (Huawei P20 Lite, 2018, China; Chipset Huawei Kirin 659; 4x2.36 GHz Cortex-A53 & 4x1.7 GHz Cortex-A53; 4GB RAM) placed with an elastic belt between the iliac crest and the great trochanter; (ii) the unstable sitting test was used to measure trunk postural control while trying to keep a centre of pressure circular trajectory seated on an labile chair placed on a force platform (9287CA, Kistler®, Switzerland); and (iii) the sudden loading sitting test was used to assess the trunk’s passive and reflex response to quick external perturbations applied (in anterior, posterior and right-lateral directions) to the upper-body centre of mass with a pneumatic piston.

**Trunk muscle endurance tests:** the maximum holding times during the execution of the front bridge endurance test, the dominant side bridge endurance test, and the Biering-Sorensen test were measured to evaluate trunk flexion, lateral bending and extension endurance, respectively.

#### Whole-body dynamic balance tests.

(i) the Y-Balance test was performed to explore stability limits in single-leg stance in three directions (anterior, posteromedial, and posterolateral); (ii) the single-leg triple hop test was used to assess the jumping performance in a single-leg power task with high balance demands; and (iii) the tandem stance and the single-leg stance posturographic tests were used to measure the whole-body dynamic balance through circular tracking tasks while standing on a force platform (9286AA, Kistler, Winterthur, Switzerland). The Y-Balance test, the single-leg stance balance test and the triple hop test were performed with both limbs (preferred and non-preferred limb), whilst in the tandem stance balance test participants placed their preferred foot ahead of the other. A brief familiarization of three repetitions per leg was performed in the Y-Balance test and in the triple hop test.

### Bridging and bird-dog exercise interventions

In the sports context, the volume at which bridging and bird-dog exercises are implemented is not usually very high as they are often integrated into general training regimens (e.g., strength, balance, plyometric exercises…) and/or included in warming-up or cooling down sessions [16,17]. For this reason, each bridging and bird-dog exercise program duration was set at 6 weeks, with 2 weekly sessions spaced 48 h apart (the entire program was held in a university fitness facility). In each of the 12 sessions, participants in the experimental groups performed four repetitions of the front bridge, back bridge, right side bridge, left side bridge and bird-dog exercises at an intensity level corresponding to the group they belonged to (i.e., EG_HI_ or EG_HV_), which was established based on the lumbopelvic accelerations recorded during the execution of the lumbopelvic postural control tests in the pre-test (Fig 1). In this sense, for each of the five exercises, the participants in the EG_HI_ performed the exercise variation (out of the seven recorded) in which lumbopelvic accelerations between 0.40 and 0.50 m/s2 were obtained at the pre-test, whilst the participants in the EG_HV_ performed the exercise variation in which pelvic accelerations between 0.20 and 0.30 m/s2 were recorded at the pre-test. These lumbopelvic acceleration ranges were established based on the lumbopelvic acceleration thresholds (cut-off point around 0.3) proposed by Heredia-Elvar et al. [18] i.e., for the EG_HI_ and the EG_HV_ each exercise variation was performed at an intensity above (0.40–0.50 m/s^2^) or below (0.20–0.30 m/s^2^) these thresholds, respectively. Regarding the training volume, whilst each repetition of the bridging and bird-dog exercise variations lasted 30 s in the EG_HV_, it lasted 15 s in the EG_HI_, resulting in one experimental group with higher volume and lower intensity (EG_HV_) and another one with higher intensity and lower volume (EG_HI_). Both groups rested 1 min between each exercise and 30 s between repetitions, which results in approximately 26.5 and 21.5 min of session duration for the EG_HV_ and the EG_HI_ group, respectively. This session duration included a 5-minute warm-up previously described by Heredia-Elvar et al. [18].

**Fig 2:**
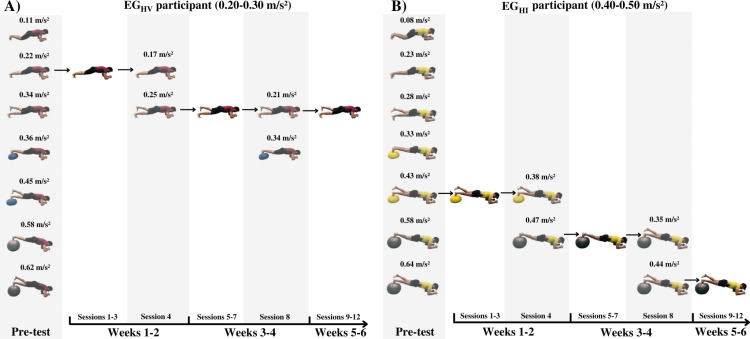
Example of the exercise intensity readjustment during the training process for two participants. The grey shaded areas represent the acceleration assessment periods. The pre-test refers to the 2nd week of baseline assessment in which all the exercise variations were performed by the participants. From all the variations, the one that was within the corresponding acceleration range of each group (i.e., 0.20-0.30 m/s^2^ for EG_HV_ and 0.40-0.50 m/s^2^ for EG_HI_) in the pre-test was selected for weeks 1 and 2 of the training period. In the 4th and 8th session, an exercise intensity readjustment was performed using the smartphone-accelerometer. In these sessions, the participants performed 2 repetitions (out of 4 for each bridging and bird-dog exercise) using the variation that they had been performing the last three sessions, and 2 repetitions using the variation that obtained the next higher lumbopelvic acceleration during the pre-test. Of both variations, the one that fell within the corresponding acceleration range of each group (i.e., 0.40–0.50 m/s^2^ or 0.20-0.30 m/s^2^) was selected as the new exercise variation for each participant. The variation with the lowest acceleration was chosen in case there was more than one variation between that range.

Lumbopelvic accelerations were recorded in the fourth and the eighth training session to adjust the intensity of each exercise throughout both 6-week bridging and bird-dog exercise programs. In these sessions, the first two repetitions of each bridging and bird-dog exercises were carried out using the exercise variation performed during the previous three sessions, and the last two repetitions were performed using the variation that obtained the following highest lumbopelvic acceleration during the pre-test (see an example of these procedures for each experimental group in Fig 2). Of both variations, the one that fell within the corresponding acceleration range of each group (i.e., 0.40–0.50 m/s^2^ or 0.20–0.30 m/s^2^) was selected as the new exercise variation for each participant. The variation with the lowest acceleration was chosen in case there was more than one variation between that range. The exercise programs were conducted and supervised by two researchers with expertise in bridging and bird-dog exercise programs that were not involved in the assessment sessions.

### Data reduction and study outcomes

For the lumbopelvic postural control tests, the two most challenging variations (i.e., the two variations with the highest acceleration scores) for each of the bridging and bird-dog exercises performed by each participant were averaged. This approach was used to reduce the large number of acceleration parameters obtained from the 28 variations of the bridging and bird-dog exercises (4 exercises × 7 variations). Lumbopelvic accelerations (m·s^−2^) were recorded at a sampling rate of 200 Hz, and the mean acceleration was calculated across the three axes (vertical, anterior-posterior, and medial-lateral). The signal was processed using a 4th-order Butterworth low-pass filter with a cut-off frequency of 10 Hz and zero-phase lag. To account for the non-stationarity of the signal, the initial and final seconds of each 15-second trial were excluded from the analysis [19]. In a stationary state, the accelerometer embedded in the smartphone exhibited a systematic error of 0.026 m/s^2^ and a random error of 0.009 m/s^2^ (95% confidence intervals). These values correspond to less than 5% and 1%, respectively, of the accelerations recorded during the lumbopelvic control tests. For participants’ trunk postural control during the unstable sitting test, as well as their whole-body dynamic balance during the tandem stance and single-leg stance posturographic tests, the center of pressure time-series were subsampled at a rate of 20 samples per second and processed using a 4th-order, zero-phase-lag Butterworth low-pass filter with a 5 Hz cut-off frequency [20], as the center of pressure signal components above 10 Hz have minimal physiological relevance [21]. To mitigate non-stationarity effects at the start of each trial, the initial 10 seconds were excluded from the analysis. To quantify performance on these tests the mean radial error was calculated as the magnitude of the vector distance (mm) between the center of pressure and the target point during the tracking tasks [22]. The average of the two best attempts (i.e., the attempts with the lowest mean radial error values) was used. In the unstable sitting test, trunk responses to sudden perturbations were evaluated using a pneumatic piston attached to a harness at the center of mass of the head, arms, and trunk with a steel cable tensioner. The piston applied a load to the trunk at a pressure of 4.2 bars and a speed of 0.5 m/s. The maximal angular displacement of the trunk (°) was measured in the anterior, posterior, and lateral directions, following the method outlined by Cholewicky et al. [23]. Angular displacement was recorded 110 ms after the perturbation. All posturographic data was processed using custom software developed by our research team within the LabView 9.0 environment (v9.0, National Instruments, Austin, Texas, USA). For the Y-Balance and the single-leg triple hop tests both legs were averaged, using the two best trials and the two closest trials for further analyses, respectively. All data processing follows the protocol outlined in a previous study, where the procedure can be consulted in greater detail [11]. The data generated or analysed during this study are included in this published article [and its Supplementary information files].

### Statistical analysis

For the statistical analysis, mean and standard deviation were calculated for all the outcomes through an intention-to-treat approach. The number of participants varied per test due to our intention-to-treat analysis, as some participants did not complete all tests or had invalid data. The normality of the data and homogeneity of variance were assessed using the Shapiro-Wilk test and Levene’s test, respectively. To facilitate interpretation of the results, pre-post changes in both absolute (Δ) and relative (Δ%) values were provided, along with their 95% confidence intervals and Cohen’s d effect sizes [24,25]. Two-way mixed ANOVAs were conducted (within-subject factor: pre-post testing sessions, between-subject factor: intervention groups) to evaluate whether the experimental and control groups produced different effects on trunk stability, trunk endurance, and whole-body balance parameters. Partial eta squared (η²p) was used to assess the effect size of the ANOVAs. Paired t-tests were used to analyze pre-post changes within each study group. The standard error of measurement (SEM) was determined by calculating the standard deviation of the differences between pre- and post-values and dividing it by the square root of 2. This value was then converted into a percentage by dividing it by the mean and multiplying by 100 [26]. The JASP 0.18.3 software (Eric-Jan Wagenmakers, Department of the Psychological Methods, University of Amsterdam, Nieuwe Achtergracht 129B, Amsterdam, Netherlands) was used for all the analyses, with a significance level set at *p* < 0.05.

## Results

**Fig 3:**
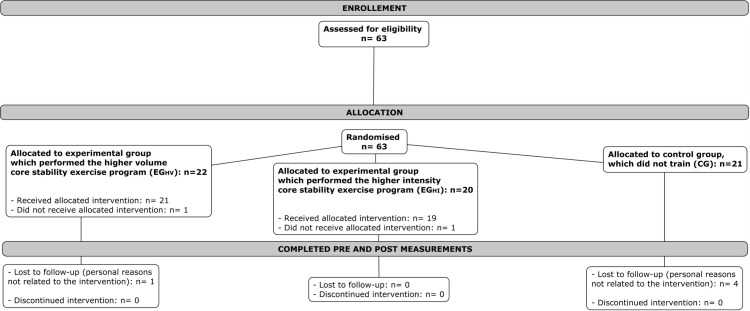
Diagram flow process of the randomized controlled trial.

Fifty-six out of the sixty-three initially recruited participants (88.9%) completed the study (Fig 3). The intervention was delivered as initially planned. Regarding the participants’ baseline characteristics (pre-test), no differences were observed for any of the variables except for the posterolateral direction of the Y-Balance test (Table 1). There was full training attendance in 82.1% of the participants, whilst the rest of participants assisted to at least 10 of the 12 training sessions; this was registered through an attendance sheet. Most participants of both experimental groups (EG_HI_: 83.3% of participants; EG_HV_: 78.9% of participants) progressed at least 1 intensity level of each exercise throughout the training program (S1 Table in S1 File). No adverse events related to this research were reported by the participants during the experimental period. Baseline characteristics of the participants at the start of the study can be seen in Table 1.

**Table 1:** Baseline characteristics and main outcomes of the participants that started the study.

	CG (n = 21)	EG_HV_ (n = 22)	EG_HI_ (n = 20)	*p* value
Age (years)	25.2 (4.6)	23.8 (4.2)	22.6 (4.9)	0.177
Height (m)	1.8 (0.1)	1.8 (0.1)	1.7 (0.1)	0.796
Weight (kg)	73.5 (8.6)	77.9 (9.3)	73.1 (6.5)	0.128
BMI (kg/m^2^)	23.8 (2.1)	24.8 (2.4)	23.6 (1.8)	0.168
***Specific trunk performance tests***				
*Bridging and bird-dog exercises stability tests – Lumbopelvic acceleration (m/s*^*2*^)				
Front bridge	0.49 (0.08)	0.51 (0.12)	0.51 (0.13)	0.779
Back bridge	0.51 (0.15)	0.57 (0.13)	0.56 (0.15)	0.448
Dominant side bridge	0.48 (0.10)	0.48 (0.10)	0.50 (0.11)	0.799
Bird-dog	0.45 (0.11)	0.49 (0.08)	0.47 (0.11)	0.452
*Trunk endurance tests – Maximal holding time (s)*				
Prone plank test	159.5 (62.2)	154.6 (48.3)	164.2 (52.1)	0.857
Dominant side bridge test	94.2 (22.6)	92.1 (22.4)	95.8 (33.2)	0.904
Biering-Sorensen test	122.2 (41.1)	120.0 (36.4)	117.3 (31.1)	0.917
***Laboratory trunk stability tests***				
*Unstable sitting test – Mean radial error (mm)*				
	7.14 (1.72)	6.95 (1.60)	6.86 (1.51)	0.856
*Sudden loading protocol – Maximal angular displacement at 110 ms (º)*				
Anterior direction	5.36 (0.92)	5.04 (0.75)	4.99 (0.73)	0.35
Lateral direction	4.59 (0.94)	4.40 (0.93)	4.55 (1.21)	0.84
Posterior direction	9.86 (1.12)	9.28 (1.20)	9.66 (1.45)	0.39
***Whole-body dynamic balance tests***				
*Y-Balance test – Distance reached normalized to the leg length (%)*				
Anterior direction	59.8 (6.6)	60.8 (5.0)	60.0 (6.3)	0.858
Posterolateral direction	102.2 (7.7)	108.2 (4.7)	104.5 (8.4)	0.034^*^
Posteromedial direction	108.5 (6.9)	110.4 (5.9)	107.7 (7.3)	0.419
Y-Balance composite	90.2 (6.6)	93.1 (4.4)	90.7 (6.9)	0.263
*Triple hop test – Distance reached normalized to the leg length (N times leg length)*				
	5.0 (0.6)	4.9 (0.7)	4.9 (0.5)	0.690
*Tandem stance balance test – Mean radial error (mm)*				
	10.05 (2.05)	10.66 (2.12)	9.46 (0.98)	0.654
*Single-leg stance balance test – Mean radial error (mm)*				
	11.38 (1.60)	11.51 (2.27)	10.94 (2.03)	0.120

Data are presented as Mean (SD). SD: standard deviation; CG: control group; EG_HV_: experimental group which performed the higher volume program; EG_HI_: experimental group which performed the higher intensity program. The initial sample differed from the indicated in the table in the following baseline outcomes: 1) Trunk acceleration during the CSE: CG = 17, EG_HV_ = 19, EG_HI_ = 19; 2) Sudden loading in anterior direction: CG = 17, EG_HV_ = 20, EG_HI_ = 18; 3) Sudden loading in lateral direction: CG = 17, EG_HV_ = 20, EG_HI_ = 18; 4) Sudden loading in posterior direction: CG = 16, EG_HV_ = 20, EG_HI_ = 17; 5) Triple hop test: CG = 14, EG_HV_ = 18, EG_HI_ = 19; 6) Single-leg and tandem balance tests: CG = 17, EG_HV_ = 19, EG_HI_ = 18. ^*^Significant pre-post differences between groups *p* < .05.

Regarding lumbopelvic postural control tests, ANOVA within subjects’ main effects showed significant changes between pre- and post-test (Table 2). Although the changes observed seem to indicate a greater impact for the experimental groups, no differences were observed between groups or *vs.* the CG. In general, there was a greater improvement tendency for the EG_HI_ with significant pre-post main changes in the lumbopelvic postural control tests (−10.4 ≤Δ% ≤−16.9) compared to the changes in the EG_HV_ (−4.8 ≤Δ% ≤−13.6). However, these pre-post changes were not significant when compared between experimental groups or with the CG. For trunk muscle endurance tests, ANOVA within subjects’ main effects showed significant changes between pre- and post-test for the experimental groups. In this sense, the EG_HV_ improved significantly in all the endurance tests (10.9 ≤Δ% ≤19.5), whilst the EG_HI_ improved only in the Biering-Sorensen test (Δ% = 15.5). Nonetheless, multiple comparisons revealed that there was no difference between experimental groups or respect to the CG.

**Table 2:** Intention-to-treat analyses of specific trunk performance tests before (pre-test) and after (post-test) the training period.

	Sample (n)	Pre-test	Post-test	Δ (LCL, UCL)	Δ % Mean (SD)	Cohen’s *d*	Interaction effect			SEM (%)
							F	*p*	η^2^p	
*Lumbopelvic postural control tests – Lumbopelvic acceleration (m/s*^*2*^)										
Front bridge	CG [21]	0.49 (0.08)	0.47 (0.08)	−0.02 (−0.06, 0.01)	−3.3 (16.5)	0.30	1.116	0.33	0.04	0.06 (12.8)
	EG_HV_ [22]	0.51 (0.12)	0.46 (0.11)	−0.05 (−0.01, −0.02)	−7.4 (20.3)*	0.49				
	EG_HI_ [20]	0.51 (0.13)	0.44 (0.09)	−0.06 (−0.02, −0.10)	−10.4 (13.5)*	0.71				
Back bridge	CG [21]	0.51 (0.15)	0.45 (0.13)	−0.06 (−0.01, −0.11)	−10.8 (16.4)*	0.56	0.437	0.65	0.01	0.09 (17.9)
	EG_HV_ [22]	0.57 (0.13)	0.48 (0.10)	−0.09 (−0.03, −0.15)	−13.6 (19.0)*	0.64				
	EG_HI_ [20]	0.56 (0.15)	0.46 (0.09)	−0.10 (−0.04, −0.16)	−15.0 (16.2)*	0.74				
Dominant side bridge	CG [21]	0.48 (0.10)	0.44 (0.09)	−0.03 (−0.08, 0.2)	−3.7 (21.1)	0.26	0.742	0.48	0.02	0.08 (17.1)
	EG_HV_ [22]	0.48 (0.10)	0.45 (0.13)	−0.03 (−0.08, 0.03)	−4.8 (23.3)	0.21				
	EG_HI_ [20]	0.50 (0.11)	0.43 (0.10)	−0.06 (−0.02, −0.11)	−11.4 (17.5)*	0.56				
Bird-dog	CG [21]	0.45 (0.11)	0.43 (0.09)	−0.02 (−0.04, 0.01)	−1.8 (12.7)	0.35	4.533	0.02*	0.13	0.06 (13.3)
	EG_HV_ [22]	0.49 (0.08)	0.45 (0.10)	−0.04 (−0.00, −0.07)	−7.3 (16.0)*	0.47				
	EG_HI_ [20]	0.47 (0.11)	0.38 (0.09)	−0.09 (−0.04, −0.14)	−16.9 (19.4)*	0.86				
*Trunk muscle endurance tests – maximal holding time (s)*										
Front bridge test	CG [20]	159.5 (62.2)	159.3 (51.9)	−0.3 (−22.0, 21.5)	7.0 (40.2)	0.01	2.413	0.09	0.09	29.9 (18.0)
	EG_HV_ [21]	154.6 (48.3)	182.7 (63.1)	28.1 (10.7, 45.5)	19.5 (25.3)*	0.74				
	EG_HI_ [19]	164.2 (52.1)	177.9 (63.4)	13.7 (−4.9, 32.4)	15.5 (26.4)	0.36				
Dominant side bridge test	CG [20]	94.2 (22.6)	92.2 (20.3)	−2.0 (−9.8, 5.9)	0.9 (21.7)	0.12	1.603	0.21	0.05	13.4 (14.1)
	EG_HV_ [21]	92.1 (22.4)	100.4 (24.3)	8.3 (1.1, 15.6)	10.9 (19.1)*	0.52				
	EG_HI_ [19]	95.8 (33.2)	97.3 (27.8)	1.4 (−9.7, 12.6)	7.1 (25.0)	0.06				
Biering-Sorensen test	CG [20]	122.2 (41.1)	120.0 (34.4)	−2.2 (−17.0, 12.6)	4.5 (30.1)	0.07	2.853	0.07	0.09	19.1 (15.3)
	EG_HV_ [21]	120.0 (36.4)	134.4 (36.3)	14.3 (6.1, 22.8)	15.1 (22.3)*	0.79				
	EG_HI_ [19]	117.3 (31.1)	132.7 (36.6)	15.4 (2.3, 28.5)	15.5 (26.4)*	0.57				

Data are presented as Mean (SD). Δ: delta of change; LCL: lower confidence limit; UCL: upper confidence limit; SD: standard deviation; SEM: standard error of measurement; CG: control group; EG_HV_: experimental group which performed the higher volume program; EG_HI_: experimental group which performed the higher intensity program. *Significant pre-post differences *p* < .05. The interaction effect reflects the ANOVA analyses (within-subject factor: pre-post testing sessions, between-subject factor: study groups). η2p: effect size in the ANOVA analysis. The confidence intervals for the Δ are set at 95%. For the lumbopelvic control tests, an average of the two most difficult variations of each exercise (i.e., the two highest accelerations) were calculated for each participant.

Regarding the laboratory trunk stability tests (Table 3), the unstable sitting test showed a significant mean radial error improvement between pre- and post-test in all groups, including the CG; however, there was not an interaction effect between them (F = 0.223; *p* = 0.801). Likewise, no significant pre-post changes nor between-group differences were observed in the sudden loading protocol.

**Table 3:** Intention-to-treat of laboratory trunk stability tests before (pre-test) and after (post-test) the training period.

	Sample (n)	Pre-test	Post-test	Δ (LCL, UCL)	Δ % Mean (SD)	Cohen’s *d*	Interaction effect			SEM (%)
							F	*p*	η^2^p	
*Unstable sitting test – Mean radial error (mm)*										
	CG [19]	7.14 (1.72)	6.72 (1.53)	−0.42 (−0.06, −0.77)	−5.0 (10.3)*	0.57	0.221	0.80	<0.01	0.56 (8.3)
	EG_HV_ [22]	6.95 (1.60)	6.44 (1.57)	−0.51 (−0.12, −0.89)	−6.9 (11.0)*	0.59				
	EG_HI_ [19]	6.86 (1.51)	6.26 (1.33)	−0.59 (−0.21, −0.97)	−7.8 (11.5)*	0.74				
*Sudden loading sitting test – Maximal angular displacement at 110 ms (º)*										
Frontal direction	CG [17]	5.36 (0.92)	5.15 (0.89)	−0.21 (−0.60, 0.19)	−3.2 (14.2)	0.27	0.891	0.42	0.03	0.67 (13.2)
	EG_HV_ [20]	5.04 (0.76)	4.91 (0.90)	−0.13 (−0.52, 0.26)	−1.8 (16.9)	0.15				
	EG_HI_ [18]	5.00 (0.73)	5.20 (1.19)	0.19 (−0.40, 0.79)	5.1 (23.3)	0.16				
Lateral direction	CG [17]	4.59 (0.94)	4.50 (1.26)	−0.09 (−0.66, 0.50)	−0.7 (23.3)	0.08	0.686	0.51	0.03	0.73 (16.3)
	EG_HV_ [20]	4.40 (0.94)	4.29 (1.06)	−0.11 (−0.61, 0.39)	−0.6 (27.4)	0.11				
	EG_HI_ [18]	4.55 (1.22)	4.81 (1.07)	0.26 (−0.24, 0.76)	9.8 (27.4)	0.26				
Posterior direction	CG [16]	9.86 (1.13)	9.41 (1.43)	−0.46 (−1.21, 0.29)	−4.1 (13.6)	0.33	0.022	0.98	<0.01	1.10 (11.8)
	EG_HV_ [20]	9.29 (1.20)	8.79 (1.58)	−0.50 (−1.36, 0.36)	−4.3 (18.0)	0.27				
	EG_HI_ [17]	9.67 (1.46)	9.28 (1.34)	−0.39 (−1.10, 0.32)	−2.9 (14.5)	0.28				

Data are presented as Mean (SD). Δ: delta of change; LCL: lower confidence limit; UCL: upper confidence limit; SD: standard deviation; SEM: standard error of measurement; CG: control group; EG_HV_: experimental group which performed the higher volume program; EG_HI_: experimental group which performed the higher intensity program. *Significant pre-post differences p < .05. The interaction effect reflects the ANOVA analyses (within-subject factor: pre-post testing sessions, between-subject factor: study groups). η2p: effect size in the ANOVA analysis. The confidence intervals for the Δ are set at 95%.

Regarding whole-body dynamic balance (Table 4), all groups showed pre-post changes in the Y-Balance test except for the anterior direction in the EG_HI_ and for the posterolateral direction in the EG_HI_ and the CG. Additionally, the EG_HI_ and the CG showed pre-post differences in the single-leg stance posturographic test. No interaction effect between-groups were observed in any of the whole-body dynamic balance tests.

**Table 4:** Intention-to-treat analyses of the whole-body dynamic balance outcomes before (pre-test) and after (post-test) the training period.

	Sample (n)	Pre-test	Post-test	Δ (LCL, UCL)	Δ %	Cohen’s *d*	Interaction effect			SEM Raw (%)
							F	*p*	η^2^p	
*Y-Balance test – Distance reached normalized to the leg length (%)*										
Anterior direction	CG [18]	59.8 (6.6)	60.9 (6.1)	1.1 (0.3, 1.9)	2.0 (2.8)*	0.70	0.235	0.79	<0.01	2.3 (3.7)
	EG_HV_ [21]	60.8 (5.0)	62.6 (4.5)	1.8 (0.2, 3.4)	3.2 (6.3)*	0.50				
	EG_HI_ [19]	60.0 (6.3)	61.3 (5.2)	1.3 (0.6, 3.2)	2.5 (7.0)	0.32				
Posterolateral direction	CG [18]	102.2 (7.7)	103.9 (6.6)	1.7 (0.1, 3.5)	1.8 (3.8)	0.47	0.221	0.80	<0.01	2.2 (2.1)
	EG_HV_ [21]	108.2 (4.7)	109.8 (5.1)	1.6 (0.5, 2.7)	1.5 (2.3)*	0.65				
	EG_HI_ [19]	104.5 (8.4)	105.6 (6.7)	1.1 (0.6, 2.7)	1.2 (3.3)	0.30				
Posteromedial direction	CG [18]	108.5 (6.9)	110.7 (5.8)	2.2 (0.8, 3.6)	2.1 (2.8)*	0.78	0.393	0.68	0.01	2.1 (1.9)
	EG_HV_ [21]	110.4 (5.9)	113.0 (5.9)	2.6 (1.0, 4.1)	2.4 (3.2)*	0.77				
	EG_HI_ [19]	107.7 (7.3)	109.4 (6.6)	1.7 (0.3, 3.1)	1.7 (2.8)*	0.58				
Composite	CG [18]	90.2 (6.6)	91.8 (5.7)	1.7 (0.7, 2.7)	1.9 (2.4)*	0.83	0.377	0.69	0.01	1.6 (1.7)
	EG_HV_ [21]	93.1 (4.4)	95.1 (4.6)	2.0 (1.0, 2.9)	2.1 (2.3)*	0.93				
	EG_HI_ [19]	90.7 (6.9)	92.1 (5.6)	1.3 (0.1, 2.6)	1.6 (3.0)*	0.50				
*Single-leg triple hop test – Distance reached normalized to the leg length (N times leg length)*										
	CG [20]	5.03 (0.58)	5.00 (0.58)	−0.03 (−0.15, 0.09)	−0.5 (5.0)	0.12	0.068	0.93	<0.01	0.2 (3.7)
	EG_HV_ [21]	4.89 (0.68)	4.89 (0.67)	0.00 (−0.10, 0.09)	0.0 (4.3)	0.01				
	EG_HI_ [19]	4.90 (0.48)	4.87 (0.46)	0.03 (−0.18, 0.13)	−0.3 (6.6)	0.09				
*Tandem stance balance posturographic test – Mean radial error (mm)*										
	CG [18]	10.05 (2.05)	9.49 (1.69)	−0.57 (−1.32, 0.19)	−4.2 (14.7)	0.37	0.343	0.71	0.01	1.3 (12.9)
	EG_HV_ [21]	10.66 (2.12)	9.94 (2.10)	−0.72 (−1.75, 0.31)	−4.6 (20.7)	0.32				
	EG_HI_ [19]	9.46 (0.98)	9.21 (1.46)	0.25 (−0.96, 0.46)	−2.0 (16.4)	0.17				
*Single-leg stance balance posturographic test – Mean radial error (mm)*										
	CG [18]	11.38 (1.60)	10.65 (1.61)	−0.73 (−1.25, −0.21)	−6.1 (9.3)*	0.70	0.437	0.65	0.02	0.9 (8.4)
	EG_HV_ [21]	11.51 (2.27)	11.09 (2.03)	−0.42 (−1.02, 0.18)	−2.9 (10.8)	0.32				
	EG_HI_ [19]	10.94 (2.03)	10.15 (1.54)	−0.78 (−1.52, −0.04)	−5.7 (14.5)*	0.51				

Data are presented as Mean (SD). Δ: delta of change; LCL: lower confidence limit; UCL: upper confidence limit; SD: standard deviation; SEM: standard error of measurement; ; CG: control group; EG_HV_: experimental group which performed the higher volume program; EG_HI_: experimental group which performed the higher intensity program. *Significant pre-post differences p < .05. The interaction effect reflects the ANOVA analyses (within-subject factor: pre-post testing sessions, between-subject factor: study groups). η2p: effect size in the ANOVA analysis. The confidence intervals for the Δ are set at 95%.

## Discussion

Although bridging and bird-dog exercises are common elements of workout routines in fitness, sports training and rehabilitation, to the best of our knowledge this is the first experimental study in young physically active male individuals that: i) analysed the impact of performing trunk-focused exercise programs in which only bridging and bird-dog exercises were performed; ii) explored the short-term effect of preforming these exercises on trunk performance and whole-body dynamic balance through a wide variety of field- and laboratory-based tests, some of which have been related to low back pain (i.e., unstable sitting and sudden loading tests) [7–10] and lower limb injury risk (i.e., Y-Balance and single-leg triple hop test) [12–14,27]; and iii) quantified, monitored and controlled the bridging and bird-dog exercise intensity through smartphone-accelerometry, which allowed the comparison of two different training doses of these exercises (EG_HI_ *vs.* EG_HV_). Based on the current study results, neither of the two 6-week bridging and bird-dog exercise programs had a significant impact on the test outcomes. Nevertheless, there was a tendency for greater and specific changes in the lumbopelvic postural control tests for the EG_HI_, and in the trunk muscle endurance tests for the EG_HV_, but these changes were not significant compared to the CG or between the experimental groups.

The lack of short-term effects of the bridging and bird-dog exercise programs confirmed our first hypothesis, supporting the results of a recent correlational study conducted on the assessments used herein [11]. That study did not find significant associations between the lumbopelvic accelerations recorded during the bridging and bird-dog exercises and the scores of the laboratory trunk stability and the whole-body dynamic balance tests in young physically active males. Overall, these findings call into question the effectiveness of using these floor-based exercises to enhance trunk stability in tasks other than those specifically used during the training (i.e., the unstable sitting and sudden loading sitting tests) or to improve whole-body balance in young male athletes without apparent postural control deficits. Other experimental studies on this population did find a significative impact on balance outcomes compared to the CG, or pre-post differences within the experimental group, but these programs combined bridging and bird-dog exercises with other trunk-focused exercises (e.g., crunches, sit-ups, back extensions, Pilates-based exercise, etc.s…) [28–30], and/or exercises that heavily engaged the lower limbs (e.g., squatting, jumps, walking lunge, frontal and lateral stance balance, shoulder contact…) [4,31]. Further research is needed to investigate the effects of performing (exclusively) bridging and bird-dog exercises on stability and postural control outcomes measured in conditions different from those of training (i.e., standing, sitting, in motion, etc.) in this population.

Contrary to our second hypothesis, no statistical differences were observed in the lumbopelvic postural control and in the trunk muscle endurance tests either between the experimental groups or with the CG (Table 2). However, from the authors’ point of view, considering the tendency observed in the magnitude of change of the experimental groups and the reduced sample size, this hypothesis cannot be clearly rejected. In this sense, both experimental groups improved the specific trunk performance outcomes significantly, with greater pre-post main changes in lumbopelvic postural control and trunk endurance for the EG_HI_ (−10.4 ≤Δ% ≤−16.9) and the EG_HV_ (10.9 ≤Δ% ≤19.5), respectively. In the case of the EG_HI_, the results show that in 3 out of 4 lumbopelvic control tests the changes reached (front bridge: Δ −0.06, SEM = 0.06) and exceeded the SEM (back bridge: Δ −0.10, SEM = 0.09; bird-dog: Δ −0.09, SEM = 0.06), indicating that the improvements go beyond the measurement error of the tests and intra-subject variability. In addition, the acceleration recording performed in the fourth and eighth training session (to adjust the bridging and bird-dog exercise intensity throughout the training programs [Fig 2]) showed a clear increase in the participants’ lumbopelvic postural control in the training exercises, which resulted in most participants (EG_HI_: 83.3% of participants; EG_HV_: 78.9% of participants) progressing in intensity at least once for each exercise (Supplementary material, S1 Table in S1 File). Research has shown that trunk-focused exercise programs significantly enhance trunk strength, stability and endurance compared to the CG [6,32,33]. However, these studies combined bridging and bird-dog exercises with other type of trunk-focused exercises (e.g., crunches, back extensions, frontal and diagonal double leg lowering…). Future studies should aim to identify which specific trunk exercises yield the greatest improvements in trunk performance.

### Limitations

There are some limitations that could bias the results interpretation. As mentioned above, the sample size was relatively small, which made it difficult to find significant differences in some outcomes, especially in those that showed greater pre-post main changes (i.e., lumbopelvic accelerations and holding times). The limited sample size resulted from extensive biomechanical testing requiring lengthy procedures, alongside the double-blind design and tailored training groups of six participants, adjusted biweekly, which required multiple research periods for recruitment. Furthermore, the short training period (i.e., 6 weeks) may have result in a low total training volume (i.e., 21.5–26.5 min of duration) and thus, these training doses for bridging and bird-dog exercises do not seem to provide a sufficient stimulus to produce significant changes in this population. Other trunk-focused exercise programs with greater volume and duration have produced significant changes compared to the CG [6,28,33], but as mentioned, these programs were more generic and did not use bridging and bird-dog exercises exclusively. Further research is needed to explore the effects of different volumes and intensities of these and other floor-based exercises in long-term training programs conducted on large samples. Finally, interpretation of these results is limited to young physically active males. In this sense, it is important to consider the participants’ physically active status, along with the fact that, although they were not following a structured training program targeting the trunk muscles at the time of the study, most of the sample had prior experience with bridging and bird-dog exercises. The inference of the results is therefore limited to this group, as, for example, trunk control in female athletes has been shown to have a greater impact on dynamic balance than in the case of men [34] and therefore, the effect of bridging and bird-dog exercises might differ from the effect observed in males. In addition, given the deficits in balance and trunk performance found in older adults [35,36] and individuals with different disorders (i.e., low back pain, stroke, multiple sclerosis…) [37–39], it would be worthwhile to examine the impact of performing bridging and bird-dog exercises on these populations.

## Conclusion

The bridging and bird-dog exercise programs performed in this study did not have a significant impact in the experimental groups compared to the CG on any of the variables analysed. In this sense, although a significant and specific pre-post intervention increase was observed in the lumbopelvic postural control tests for the EG_HI_, and in the trunk muscle endurance tests for the EG_HV_, the current results question the short-term effectiveness of these floor-based exercise programs in young physically active males. Further research is needed to explore the effects of different doses of these exercises in young individuals in order to confirm the results obtained in this study.

## Supporting information

S1 File This supporting information file contains data on the progression of participants in both experimental groups, as well as lumbopelvic acceleration measurements for all variations of the front plank, back plank, side plank, and bird-dog exercises. (DOCX)

S1 Data Database article. (XLSX)
