# Supplementary material for: Short-term bridging and bird-dog exercise programs did not enhance trunk performance and whole-body dynamic balance in young physically active males: A double-blind randomized trial
Source: PLoS One. 2025 Jun 5;20(6):e0325040. doi: 10.1371/journal.pone.0325040 (PMC12140198; doi:10.1371/journal.pone.0325040)
Supplement: S1 File — (DOCX) [file pone.0325040.s001.docx]

| **S1 Table. Percentage of participants of both experimental groups who progressed 0, 1 or 2 intensity levels in each exercise (i.e., changing to a more difficult exercise variation) during the exercise program.** | | |
| --- | --- | --- |
|  | **EG_HV_ (n=20)** | **EG_HI_ (n=19)** |
| *Front bridge* | | |
| No progression | 5.3 | 11.1 |
| Increased 1 level | 42.1 | 27.8 |
| Increased 2 levels | 52.6 | 61.1 |
| *Back bridge* | | |
| No progression | 21.1 | 5.6 |
| Increased 1 level | 36.8 | 66.7 |
| Increased 2 levels | 42.1 | 27.8 |
| *Dominant side bridge* | | |
| No progression | 10.5 | 16.7 |
| Increased 1 level | 52.6 | 38.9 |
| Increased 2 levels | 36.8 | 44.4 |
| *Bird-dog* | | |
| No progression | 15.8 | 11.1 |
| Increased 1 level | 47.4 | 55.6 |
| Increased 2 levels | 36.8 | 33.3 |
| EG_HV_: experimental group which performed the higher volume program; EG_HI_: experimental group which performed the higher intensity program. | | |

| **S2 Table. Exercise variations in which participants from each core stability exercise program trained.** | | |
| --- | --- | --- |
|  | **EG_HV_ (n=20)** | **EG_HI_ (n=19)** |
| *Frontal bridge – mean (SD)* | | |
| Weeks 1-2 | 2.4 (0.9) | 4.3 ± 1.1 |
| Weeks 3-4 | 3.4 (0.9) | 5.6 ± 1.1 |
| Weeks 5-6 | 4.3 (1.3) | 6.4 ± 1.0 |
| *Back bridge – mean (SD)* | | |
| Weeks 1-2 | 2.3 (1.5) | 4.1 (1.3) |
| Weeks 3-4 | 2.9 (1.1) | 4.4 (1.5) |
| Weeks 5-6 | 3.5 (1.3) | 5.1 (1.5) |
| *Dominant side bridge – mean (SD)* | | |
| Weeks 1-2 | 1.8 (0.7) | 3.1 (1.1) |
| Weeks 3-4 | 2.4 (0.7) | 3.9 (1.2) |
| Weeks 5-6 | 3.0 (0.8) | 4.5 (1.4) |
| *Left side bridge – mean (SD)* | | |
| Weeks 1-2 | 1.8 (0.7) | 3.1 (1.1) |
| Weeks 3-4 | 2.2 (0.7) | 3.9 (1.1) |
| Weeks 5-6 | 2.8 (0.8) | 4.3 (1.4) |
| *Bird-dog* | | |
| Weeks 1-2 | 2.8 (1.0) | 5.4 (1.3) |
| Weeks 3-4 | 3.5 (1.0) | 6.8 (1.3) |
| Weeks 5-6 | 4.2 (1.1) | 6.7 (1.1) |
| EG_HV_: experimental group which performed the higher volume program; EG_HI_: experimental group which performed the higher intensity program. | | |

| **S3 Table.** **Intention-to-treat analyses of lumbopelvic acceleration (m/s^2^) during all front bridge variations before (Pre-test) and after (Post-test) the training period.** | | | | |
| --- | --- | --- | --- | --- |
|  | **Sample (n)** | **Pre-test** | **Post-test** | **Δ (%)** |
| **A** | CG (21) | 0.09 (0.02) | 0.10 (0.05) | 14.9 (30.2)* |
|  | EG_HV_ (22) | 0.09 (0.03) | 0.09 (0.03) | -0.4 (34.3) |
|  | EG_HI_ (20) | 0.09 (0.02) | 0.09 (0.03) | 6.6 (27.4)* |
| **B** | CG (21) | 0.23 (0.11) | 0.24 (0.10) | 6.9 (25.3)* |
|  | EG_HV_ (22) | 0.23 (0.11) | 0.21 (0.10) | -7.8 (23.2)* |
|  | EG_HI_ (20) | 0.20 (0.09) | 0.21 (0.08) | 10.7 (24.8)* |
| **C** | CG (21) | 0.37 (0.09) | 0.36 (0.09) | -2.1 (12.4)* |
|  | EG_HV_ (22) | 0.36 (0.11) | 0.34 (0.10) | -2.0 (20.8)* |
|  | EG_HI_ (20) | 0.39 (0.13) | 0.36 (0.10) | -3.6 (18.0)* |
| **D** | CG (21) | 0.30 (0.15) | 0.31 (0.14) | 9.3 (28.6)* |
|  | EG_HV_ (22) | 0.32 (0.11) | 0.28 (0.12) | -9.6 (24.4)* |
|  | EG_HI_ (20) | 0.26 (0.10) | 0.26 (0.10) | 3.7 (23.6)* |
| **E** | CG (21) | 0.42 (0.10) | 0.43 (0.10) | 3.4 (18.6)* |
|  | EG_HV_ (22) | 0.41 (0.10) | 0.39 (0.12) | -6.1 (16.0)* |
|  | EG_HI_ (20) | 0.43 (0.13) | 0.41 (0.11) | -1.2 (19.3)* |
| **F** | CG (21) | 0.41 (0.11) | 0.40 (0.10) | 1.8 (24.8)* |
|  | EG_HV_ (21) | 0.41 (0.12) | 0.37 (0.12) | -9.8 (18.6)* |
|  | EG_HI_ (20) | 0.37 (0.10) | 0.34 (0.09) | -6.5 (15.5)* |
| **G** | CG (19) | 0.54 (0.08) | 0.52 (0.06) | -2.6 (14.2)* |
|  | EG_HV_ (21) | 0.56 (0.16) | 0.52 (0.13) | -3.4 (24.9)* |
|  | EG_HI_ (20) | 0.56 (0.15) | 0.49 (0.09) | -10.2 (15.7)* |
| Data are presented as Mean (SD). Δ (%): relative delta of change; SD: standard deviation; CG: control group; EG_HV_: experimental group which performed the higher volume program; EG_HI_: experimental group which performed the higher intensity program. *Significant pre-post differences *p*<.05. | | | | |

| **S4 Table. Intention-to-treat analyses of lumbopelvic acceleration (m/s^2^) during all back bridge variations before (Pre-test) and after (Post-test) the training period.** | | | | |
| --- | --- | --- | --- | --- |
|  | **Sample (n)** | **Pre-test** | **Post-test** | **Δ (%)** |
| **A** | CG (21) | 0.14 (0.05) | 0.13 (0.07) | -3.1 (18.5)* |
|  | EG_HV_ (22) | 0.16 (0.05) | 0.16 (0.06) | -3.2 (32.5)* |
|  | EG_HI_ (20) | 0.15 (0.05) | 0.15 (0.05) | 4.6 (31.7)* |
| **B** | CG (21) | 0.42 (0.14) | 0.38 (0.13) | -8.3 (10.7)* |
|  | EG_HV_ (22) | 0.44 (0.15) | 0.40 (0.14) | -6.8 (23.1)* |
|  | EG_HI_ (20) | 0.43 (0.17) | 0.39 (0.12) | -5.8 (21.0)* |
| **C** | CG (21) | 0.20 (0.06) | 0.21 (0.08) | 5.6 (40.7) |
|  | EG_HV_ (22) | 0.24 (0.08) | 0.20 (0.06) | -12.8 (28.2)* |
|  | EG_HI_ (20) | 0.22 (0.09) | 0.22 (0.08) | 3.0 (24.4)* |
| **D** | CG (21) | 0.42 (0.14) | 0.38 (0.14) | -8.0 (21.8)* |
|  | EG_HV_ (22) | 0.44 (0.17) | 0.39 (0.13) | -6.1 (27.7)* |
|  | EG_HI_ (20) | 0.42 (0.15) | 0.40 (0.12) | -0.8 (22.4)* |
| **E** | CG (19) | 0.38 (0.10) | 0.36 (0.11) | -3.4 (24.6)* |
|  | EG_HV_ (21) | 0.44 (0.15) | 0.36 (0.09) | -14.0 (23.0) |
|  | EG_HI_ (18) | 0.43 (0.12) | 0.33 (0.08) | -18.8 (20.3) |
| **F**^†^ | CG (18) | 0.52 (0.11) | 0.51 (0.12) | -1.5 (14.8)* |
|  | EG_HV_ (20) | 0.54 (0.10) | 0.53 (0.09) | 0.8 (23.7) |
|  | EG_HI_ (18) | 0.55 (0.09) | 0.46 (0.08) | -14.8 (10.6)* |
| **G** | CG (6) | 0.63 (0.29) | 0.46 (0.16) | -20.5 (26.4) |
|  | EG_HV_ (10) | 0.55 (0.16) | 0.49 (0.12) | -5.6 (25.7) |
|  | EG_HI_ (11) | 0.59 (0.26) | 0.52 (0.12) | -5.6 (22.8)* |
| Data are presented as Mean (SD). Δ (%): relative delta of change; SD: standard deviation; CG: control group; EG_HV_: experimental group which performed the higher volume program; EG_HI_: experimental group which performed the higher intensity program. *Significant pre-post differences *p*<.05. ^†^Significantly differences between groups. | | | | |

| **S5 Table.** **Intention-to-treat analyses of lumbopelvic acceleration (m/s^2^) during all dominant side bridge variations before (Pre-test) and after (Post-test) the training period.** | | | | |
| --- | --- | --- | --- | --- |
|  | **Sample (n)** | **Pre-test** | **Post-test** | **Δ (%)** |
| **A** | CG (21) | 0.18 (0.06) | 0.18 (0.07) | -3.9 (23.3)* |
|  | EG_HV_ (22) | 0.17 (0.05) | 0.16 (0.05) | 1.6 (26.3)* |
|  | EG_HI_ (20) | 0.19 (0.06) | 0.16 (0.05) | -12.1 (23.6) |
| **B** | CG (21) | 0.33 (0.10) | 0.32 (0.10) | 0.3 (23.3)* |
|  | EG_HV_ (22) | 0.31 (13) | 0.31 (0.09) | 8.8 (33.7)* |
|  | EG_HI_ (20) | 0.33 (0.12) | 0.29 (0.07) | -7.6 (21.2)* |
| **C** | CG (21) | 0.39 (0.09) | 0.40 (0.09) | 6.5 (23.3)* |
|  | EG_HV_ (22) | 0.40 (0.12) | 0.40 (0.11) | 5.3 (31.4)* |
|  | EG_HI_ (20) | 0.42 (0.10) | 0.40 (0.10) | -4.9 (17.2)* |
| **D** | CG (21) | 0.39 (0.09) | 0.40 (0.10) | 4.4 (21.9)* |
|  | EG_HV_ (22) | 0.37 (0.10) | 0.39 (0.11) | 15.0 (63.4) |
|  | EG_HI_ (20) | 0.40 (0.13) | 0.37 (0.10) | -3.8 (19.0)* |
| **E** | CG (19) | 0.46 (0.11) | 0.46 (0.10) | 2.2 (20.8) |
|  | EG_HV_ (21) | 0.46 (0.11) | 0.46 (0.13) | 5.5 (43.9)* |
|  | EG_HI_ (18) | 0.48 (0.12) | 0.44 (0.11) | -6.3 (13.5)* |
| **F** | CG (9) | 0.57 (0.13) | 0.48 (0.09) | -12.8 (21.3) |
|  | EG_HV_ (13) | 0.52 (0.09) | 0.48 (0.18) | -6.2 (29.4) |
|  | EG_HI_ (12) | 0.47 (0.15) | 0.42 (0.13) | -7.1 (22.5)* |
| Data are presented as Mean (SD). Δ (%): relative delta of change; SD: standard deviation; CG: control group; EG_HV_: experimental group which performed the higher volume program; EG_HI_: experimental group which performed the higher intensity program. *Significant pre-post differences *p*<.05. | | | | |

| **S6 Table.** **Intention-to-treat analyses of lumbopelvic acceleration (m/s^2^) during all bird-dog bridge variations before (Pre-test) and after (Post-test) the training period.** | | | | |
| --- | --- | --- | --- | --- |
|  | **Sample (n)** | **Pre-test** | **Post-test** | **Δ (%)** |
| **A** | CG (21) | 0.12 (0.04) | 0.12 (0.05) | -1.3 (19.4)* |
|  | EG_HV_ (22) | 0.14 (0.04) | 0.14 (0.05) | -1.5 (28.8)* |
|  | EG_HI_ (20) | 0.14 (0.06) | 0.13 (0.04) | -4.6 (16.4)* |
| **B** | CG (21) | 0.16 (0.05) | 0.16 (0.05) | 4.5 (21.9)* |
|  | EG_HV_ (22) | 0.17 (0.04) | 0.17 (0.05) | 0.8 (25.4)* |
|  | EG_HI_ (20) | 0.17 (0.06) | 0.16 (0.04) | -2.5 (17.9)* |
| **C**^†^ | CG (21) | 0.23 (0.08) | 0.25 (0.08) | 13.1 (34.0)* |
|  | EG_HV_ (22) | 0.24 (0.07) | 0.23 (0.09) | -4.0 (33.2)* |
|  | EG_HI_ (20) | 0.23 (0.08) | 0.20 (0.06) | -13.0 (16.9)* |
| **D** | CG (21) | 0.20 (0.06) | 0.20 (0.07) | 2.8 (32.2)* |
|  | EG_HV_ (22) | 0.26 (0.09) | 0.22 (0.09) | -11.0 (22.2)* |
|  | EG_HI_ (20) | 0.24 (0.11) | 0.20 (0.08) | -9.5 (28.3)* |
| **E** | CG (21) | 0.39 (0.10) | 0.38 (0.09) | 2.7 (20.8)* |
|  | EG_HV_ (22) | 0.44 (0.09) | 0.41 (0.11) | -6.1 (16.3)* |
|  | EG_HI_ (20) | 0.39 (0.13) | 0.34 (0.10) | -10.1 (22.3)* |
| **F** | CG (21) | 0.34 (0.10) | 0.33 (0.11) | -1.9 (19.7)* |
|  | EG_HV_ (22) | 0.37 (0.10) | 0.34 (0.09) | -6.0 (16.2)* |
|  | EG_HI_ (20) | 0.33 (0.10) | 0.31 (0.08) | -5.9 (14.9)* |
| **G**^†^ | CG (21) | 0.50 (0.14) | 0.50 (0.11) | 13.6 (58.3)* |
|  | EG_HV_ (20) | 0.54 (0.09) | 0.50 (0.10) | -7.5 (17.4)* |
|  | EG_HI_ (19) | 0.52 (0.13) | 0.41 (0.09) | -17.6 (22.8) |
| Data are presented as Mean (SD). Δ (%): relative delta of change; SD: standard deviation; CG: control group; EG_HV_: experimental group which performed the higher volume program; EG_HI_: experimental group which performed the higher intensity program. *Significant pre-post differences *p*<.05. ^†^Significantly differences between groups. | | | | |
